# Nonenzymatic Serum Antioxidant Capacity in IBD and Its Association with the Severity of Bowel Inflammation and Corticosteroids Treatment

**DOI:** 10.3390/medicina55040088

**Published:** 2019-04-02

**Authors:** Katarzyna Neubauer, Radoslaw Kempinski, Malgorzata Matusiewicz, Iwona Bednarz-Misa, Malgorzata Krzystek-Korpacka

**Affiliations:** 1 Department of Gastroenterology and Hepatology, Wroclaw Medical University, 50-556 Wroclaw, Poland; radoslaw.kempinski@umed.wroc.pl; 2 Department of Medical Biochemistry, Wroclaw Medical University, 50-368 Wroclaw, Poland; malgorzata.matusiewicz@umed.wroc.pl (M.M.); iwona.bednarz-misa@umed.wroc.pl (I.B.-M.); malgorzata.krzystek-korpacka@umed.wroc.pl (M.K.-K.)

**Keywords:** free thiols, uric acid, total antioxidant status, inflammatory bowel disease (IBD), Crohn’s disease, ulcerative colitis, mucosal healing

## Abstract

*Background and objectives*: Oxidative stress signalling plays a monumental role in inflammatory bowel disease (IBD). Reduction of oxidative stress might control inflammation, block tissue damage, and reverse natural history of IBD. We assessed the serum concentrations of free thiols (FT) and uric acid (SUA), together constituting a large part of nonenzymatic serum antioxidant capacity, as well as total antioxidant status (TAS) with reference to IBD phenotype, activity, co-occurrence of anemia, and treatment with azathioprine (AZA) and corticosteroids (CS). Additionally, we appraised the potential of uric acid, thiol stress, and TAS as mucosal healing (MH) markers in ulcerative colitis. *Materials and methods*: SUA, FT, and TAS were measured colorimetrically using, respectively, uricase, Ellman’s and 2,2′-azino-bis-3-ethylbenzthiazoline-6-sulphonic acid (ABTS) methods. *Results*: The study group consisted of 175 individuals: 57 controls, 71 ulcerative colitis (UC), and 47 Crohn’s disease (CD) patients. When compared to controls, SUA levels were significantly lower in patients with CD, and FT and TAS levels were significantly lower in patients with CD and UC. In UC patients, SUA, FT, and TAS inversely correlated with the severity of bowel inflammation. As MH markers, SUA displayed better overall accuracy and higher specificity than FT. In active CD, FT, and SUA were significantly lower in patients with anemia. FT was significantly lower in patients treated with corticosteroids. *Conclusions*: IBD patients, regardless the disease phenotype, have systemic thiol stress, depleted total antioxidant capacity, and reduced concentrations of uric acid, reflecting, to various degrees, clinical and local disease activity as well as presence of anaemia, the most common extraintestinal manifestation of IBD. Evaluation of systemic total antioxidant status may be useful in noninvasive assessment of mucosal healing. Our findings on thiol stress provide an additional aspect on adverse effects of corticosteroids therapy.

## 1. Introduction

Inflammatory bowel disease (IBD) is a broad term describing complex, systemic, and incurable diseases encompassing Crohn’s disease (CD) and ulcerative colitis (UC). IBD affects people all over the world with the strong growing trend in incidence rates. For instance, 1.6 million Americans currently have an IBD diagnosis and there are 70,000 new cases diagnosed each year. Unquestionable progress in searching for the mechanisms of underlying pathogenesis of IBD has led to a generally accepted concept, that it is a result of interaction between immune system and gut microbiota in genetically susceptible individuals in presence of environmental factors [1]. Still, other unresolved and challenging issues related with CD and UC include a complicated diagnostic and monitoring process as well as unsatisfactory therapeutic options with total effectiveness lower than 50% [2]. Mucosal healing (MH) has recently been recognized as a key objective in the management of IBD patients. Endoscopy, CT, and MRI are employed for the accurate evaluation of intestinal healing. However, these techniques are either invasive or expensive and as such not optimal for regular monitoring of IBD patients [3]. Therefore, surrogate markers, noninvasive, easily measured, and inexpensive, are needed and intensively looked for [4].

Oxidative stress, defined as an imbalance between prooxidants and antioxidants, is a key player in the mechanism underpinning mucosal damage in IBD. Oxidative stress has been implicated in the propagation and exacerbation, if not pathogenesis, of IBD as the defective antioxidant system in IBD may be a consequence of genetic variants [5]. Moreover, it has been repeatedly shown that IBD-related oxidative stress is not restricted to the intestinal mucosa but manifests itself at the systemic level as well [6]. Accordingly, our group has previously demonstrated both the accumulation of markers of oxidative damage to macromolecules [7,8] and the depletion of enzymatic antioxidant defenses [9,10] in IBD. Additionally, antioxidant responses are affected in patients with IBD and concomitant primary sclerosing cholangitis, being fatal extraintestinal manifestations of the disease [11].

In this study, we assessed the serum concentrations of free thiols (FT) and uric acid (SUA), constituting together a large part of nonenzymatic serum antioxidant capacity, as well as total antioxidant status (TAS) with reference to IBD phenotype, activity, co-occurrence of anemia, as an extraintestinal manifestation of IBD, and treatment with azathioprine (AZA) and corticosteroids (CS). Additionally, we appraised the potential of uric acid, thiol stress, and TAS as MH markers in ulcerative colitis.

## 2. Materials and Methods

### 2.1. Patients

The study group consisted of consecutive IBD patients admitted to the Department of Gastroenterology and Hepatology of Wroclaw Medical University due to either a disease flare-up or for monitoring and controls. Patients with indeterminate colitis or coexistence of other severe systemic diseases, diabetes, renal failure, celiac disease, hypo- and hyperthyroidism, malignancies, liver diseases, or pregnancies were not included. The control group consisted of the healthy subjects recruited from the hospital staff.

For the assessment of CD activity, the Crohn’s Disease Activity Index (CDAI), combining the evaluation of vital parameters, clinical findings, and medical history (as described in detail elsewhere [12]) was used. For the assessment of UC activity, the Clinical Activity Index (CAI), also known as Rachmilewitz Index (RI), encompassing stool frequency, number of stools with blood, general well-being, abdominal pain/cramps, fever, extraintestinal manifestations, and laboratory tests: erythrocyte sedimentation rate (ESR) and hemoglobin concentration was used. Endoscopic findings in UC were evaluated according to the Mayo Scoring System for Assessment of Ulcerative Colitis Activity [13] as follows: a score of 0 was allocated for inactive disease, 1 for mild disease (erythema, decreased vascular pattern, mild friability), 2 for moderate disease (marked erythema, lack of vascular pattern, friability, erosions), and 3 for severe disease (spontaneous bleeding, ulceration). Mucosal healing was defined as absence of friability, blood, erosions, and ulcers of the gut mucosa [4].

Anemia was defined according to WHO criteria as hemoglobin level <12 g/dL in women and <13 g/dL in men. Transferrin concentrations were used as a surrogate marker of nutritional status. All patients declared consumption of the standard diet and denied consumption of dietary supplements. They also declared themselves as nondrinking or drinking alcohol only occasionally and in small amounts. Detailed characteristics of study and control groups are given in Table 1.

### 2.2. Analytical Methods

Blood, in a fasting state, was drawn by venous puncture and collected into serum-separator tubes, clotted, and centrifuged (30’, 720× *g*). Serum samples were stored at −80 °C until examination.

Uric acid (SUA) concentrations were measured with uricase method using SmartTest Diagnostics Uric Acid Reagent Set (Orgenics Ltd., Courbevoie, France). The uricase method is based on SUA conversion into allantoin and hydrogen peroxides, which initiates the coupling of 4-aminoantipyrine to 3,5-dichloro-2-hydroxybenzene sulfonic and leads to the formation of chromogen, measured at 520 nm.

The concentrations of free thiols (FT) were determined colorimetrically at 405 nm with Ellman’s method [14] adapted to microtiter plates, using 5,5′-dithiobis (2-nitrobenzoic acid) (DTNB) from Sigma-Aldrich (SaintLouis, MO, USA). Standard curve was prepared with reduced glutathion (Sigma-Aldrich) as a calibrator within 0–1000 µmol/L range.

Total Antioxidant Status (TAS) kits purchased from Randox Laboratories Ltd. (Crumlin, UK) were applied for the assessment of the overall serum antioxidant capacity. It is based on the suppression of the formation of 2,2′-azinobis (3-ethylbenzothiazoline-6-sulfonate; ABTS*^+^), mediated mainly by the subsequent antioxidants: uric acid, protein thiol groups, ascorbic acid, and tocopherol. All measurements were conducted in duplicate and technical replicates were averaged prior to statistical analysis.

For the purpose of correlation analysis, data on interleukins (IL)-1 and -6 and tumor necrosis factor (TNF)-α, (measured by an enzyme double-antibody indirect immunoassays with PeliKine Compact human IL-1, IL-6, and TNF- α ELISA kits from Sanquin, Amsterdam, The Netherlands), on iron concentrations (measured colorimetrically using chromazurol B method in a presence of lipid clearing factor (LCF) with an assay provided by Emapol, Gdansk, Poland), on advanced oxidation protein products (AOPP; measured using the spectrophotometric method of Witko-Sarsat et al. [15]), on transferrin concentrations (measured using the enhanced immunoturbidimetric method with an assay provided by Stamar, Dabrowa Gornicza, Poland), or on high-sensitive C-reactive protein (hsCRP; determined by the latex particle-enhanced immunoturbidimetric method with the CRPex-HS CRP test from Good Biotech Corp., Taichung, Taiwan, with protein multicalibrator from ProDia International, Sharjah, UAE as a standard), were retrieved from our database. [7,16] Other laboratory indices, such as erythrocyte sedimentation rate (ESR), thrombocytes’ count (PLT), hemoglobin concentration (Hb), aminotransferases, and creatinine were assessed with routine automatic procedures.

### 2.3. Statistical Analysis

Data distribution was tested using Kolmogorov–Smirnov test and homogeneity of variances using Levene’s test. Data were analyzed using Mann–Whitney U test or Kruskal–Wallis H tests with Conover post-hoc test or with one-way ANOVA. Multivariate analysis (stepwise method) was used to allow for age-, sex-, the disease duration-, smoking and nutritional status-adjusted analysis (data were log-transformed prior to analysis to assure normality of distribution and homogeneity of variances). Correlation analysis was conducted using Spearman correlation test and frequency analysis using either Fisher’s exact test (2 × 2) or Chi-square test. Receiver Operating Characteristics (ROC) curve analysis was conducted to evaluate nonenzymatic serum antioxidants as biomarkers in IBD. The area under the ROC curves (AUC), expressed in %, represented overall diagnostic accuracy of evaluated marker. Additionally, marker’s sensitivity and specificity corresponding with an optimal cut-off value were calculated. All probabilities were two-sided and *p* ≤ 0.05 was considered statistically significant.

All statistical analyses were conducted using MedCalc Statistical Software version 18.6 (MedCalc Software bvba, Ostend, Belgium; http://www.medcalc.org; 2018).

### 2.4. Ethical Considerations

The study protocol was approved by the Medical Ethics Committees of Wroclaw Medical University (KB-71/2007) and the study was conducted in accordance with the Helsinki Declaration of 1975, as revised in 1983, and informed consent was obtained from all patients.

## 3. Results

### 3.1. Patients

The study group consisted of 175 individuals: 57 controls and 118 consecutive IBD patients (71 with ulcerative colitis (UC) and 47 with Crohn’s disease (CD)). Almost all IBD patients were treated with 5′-aminosalicylate (5′-ASA) derivatives; 43 patients were treated with corticosteroids and 34 with azathioprine. Only one patient was taking infliximab. Of other medications, three patients were taking metronidazole, five ciprofloxacin, and two—both metronidazole and ciprofloxacin.

### 3.2. Nonenzymatic Serum Antioxidants in IBD

**Figure 1:**
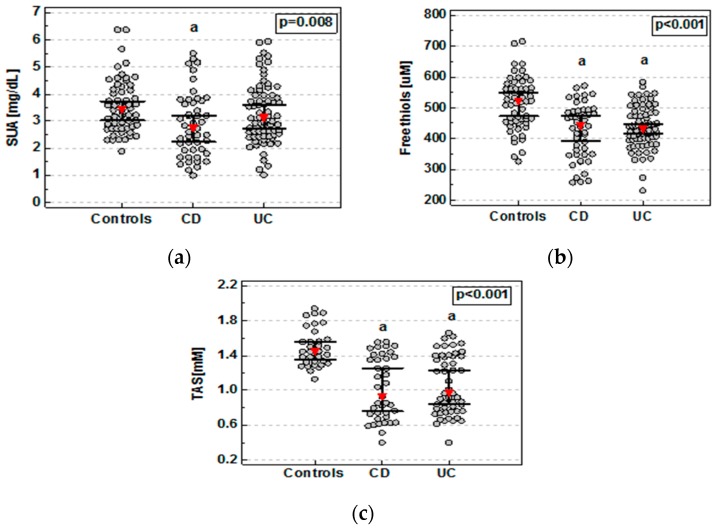
Serum uric acid (**a**), plasma free thiol status (**b**), and total antioxidant status (**c**) in inflammatory bowel disease (IBD) patients.

**Figure 2:**
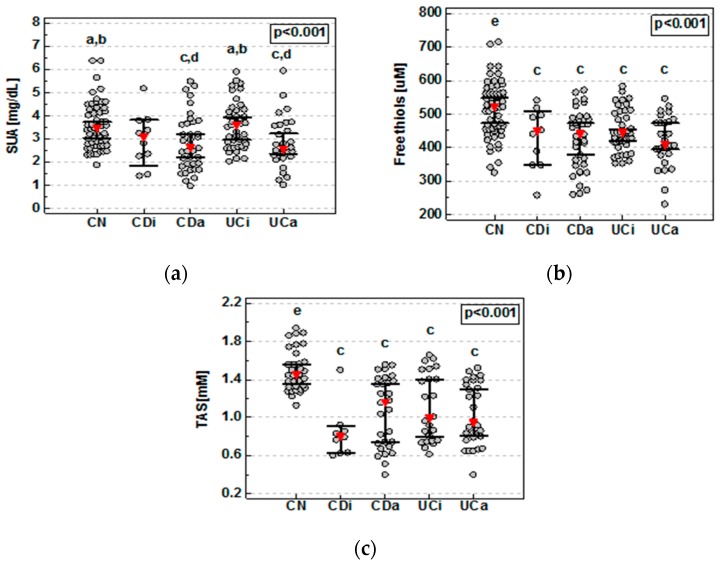
Effect of the disease activity on serum uric acid (**a**), plasma free thiol status (**b**), and total antioxidant status (**c**).

As compared to controls, SUA concentrations were significantly lower in patients with CD but not UC (Figure 1a). Detailed analysis demonstrated that SUA concentrations were significantly lower in both active CD and UC. These patients also had significantly lower levels of SUA compared to patients with inactive UC (Figure 2a). SUA association with active IBD remained significant (*p* < 0.0001) following the adjustment to age, sex (*p* < 0.0001), smoking status, and transferrin. In CD patients with active disease, SUA concentrations inversely correlated with CDAI (ρ = −0.35, *p* = 0.038). There was no significant correlation between SUA and RI in patients with active UC.

Compared to controls, FT concentrations were significantly lower in patients with both CD and UC (Figure 1b), regardless of the disease activity (Figure 2b). FT association with IBD remained significant (*p* < 0.0001) following the adjustment for age, sex, smoking status, and transferrin (*p* = 0.003). There were no significant differences between CD and UC or patients with active and inactive disease. However, in CD and UC patients with active disease, FT concentrations were inversely correlated with, respectively, CDAI (ρ = −0.52, *p* = 0.001) and RI (ρ = −0.40, *p* = 0.044). FT concentrations remained lower in both CD (*p* = 0.023) and UC (*p* = 0.002) as compared to controls also following adjustment to albumin concentrations.

Serum samples for TAS assessment were available for 123 individuals: 33 controls, 39 CD, and 51 UC. TAS was significantly reduced in both CD and UC patients as compared to healthy controls (Figure 1c) without significant differences between both disease phenotypes or with respect to the disease activity (Figure 2c). TAS association with IBD remained significant (*p* < 0.0001) following the adjustment for age, sex, smoking status, and transferrin. The inverse relation between TAS and CDAI or RI in IBD patients with active disease did not reach statistical significance (ρ = −0.34, *p* = 0.123 and ρ = −0.33, *p* = 0.166).

There was no correlation between any of the examined antioxidants and the disease duration, the activities of aminotransferases, or creatinine concentrations either in the whole cohort or in phenotype- or activity-based subgroups.

Data was analyzed using Kruskal–Wallis H test with Conover post-hoc test. Red triangles represent median and whiskers 95% CI. CD, Crohn’s disease; UC, ulcerative colitis; a, significantly different from controls.

Data was analyzed using the Kruskal–Wallis H test with Conover post-hoc test. Red triangles represent median and whiskers 95% CI. CDa, active Crohn’s disease; CDi, inactive Crohn’s disease; CN, controls; UCa, active ulcerative colitis; UCi, inactive ulcerative colitis; a, significantly different from CDa; b, significantly different from UCa; c, significantly different from CN; d, significantly different from UCi; e, significantly different from all other groups.

### 3.3. Nonenzymatic Serum Antioxidants and Mucosal Healing (MH)

**Figure 3:**
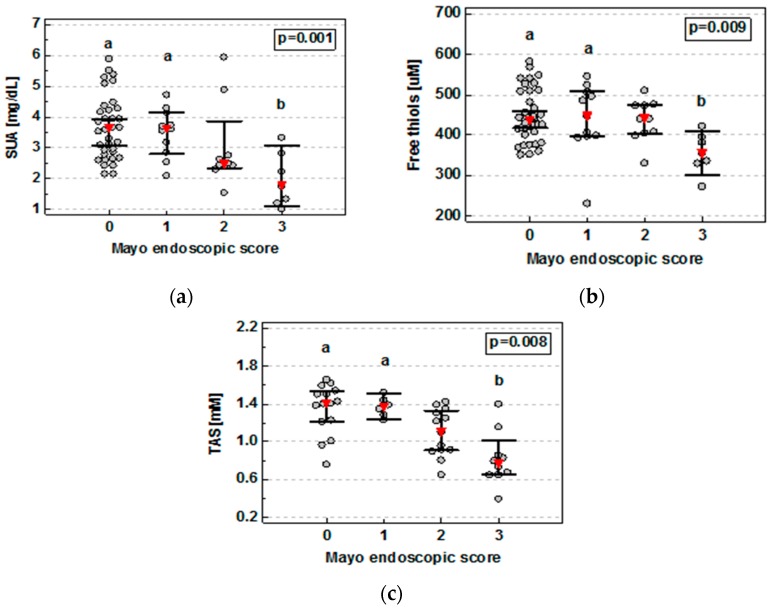
Serum uric acid (**a**), plasma free thiol status (**b**), and total antioxidant status (**c**) correlation with Mayo endoscopic score in ulcerative colitis patients.

In UC patients, SUA (ρ = −0.41, *p* < 0.001) and FT (ρ = −0.27, *p* = 0.023) concentrations as well as TAS (ρ = −0.58, *p* = 0.002) were inversely correlated with the severity of bowel inflammation expressed in terms of Mayo endoscopic score with a significant drop between score 3 and scores 0 and 1 (Figure 3).

Data was analyzed using Kruskal–Wallis H test with Conover post-hoc test. Red triangles represent median and whiskers 95% CI. a, significantly different from score 3; b, significantly different from scores 0 and 1.

**Figure 4:**
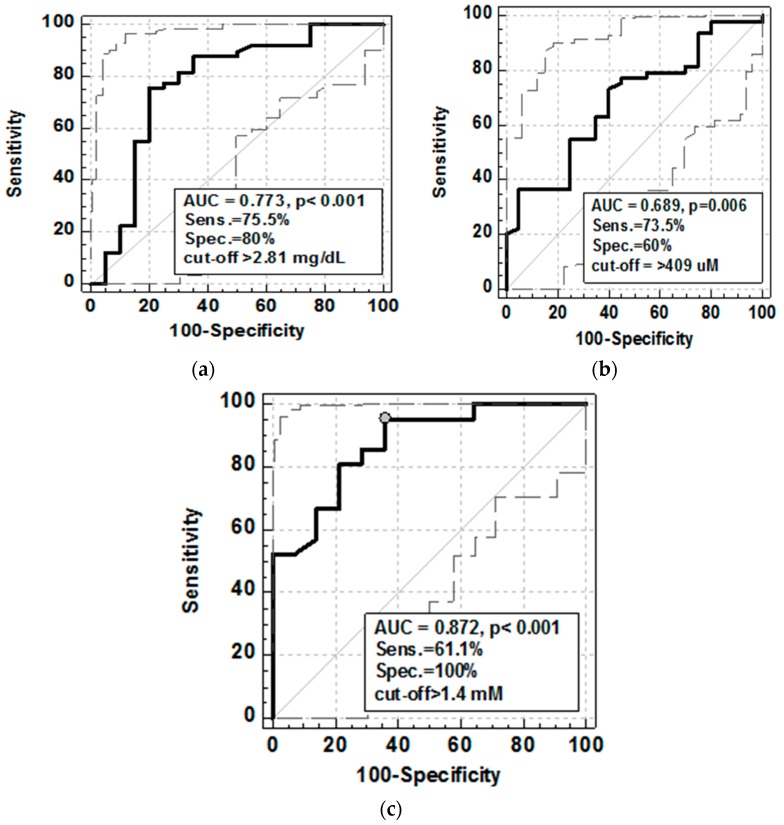
Serum uric acid (**a**), plasma free thiol status (**b**), and total antioxidant status (**c**) as mucosal healing markers in ulcerative colitis patients. Data presented as Receiver Operating Characteristics (ROC) curve with 95% CI (respectively straight and dashed lines). AUC, area under ROC curve; Sens., sensitivity; Spec., specificity.

To evaluate the strength of the association as well as the potential of SUA, FT, and TAS as the MH markers, ROC analysis was employed. Mucosal healing was defined as the Mayo score drop to 0 or 1 [3]. As the MH markers, SUA displayed better overall accuracy, expressed in terms of area under ROC curve, and higher specificity than FT. The accuracy and specificity of TAS was superior to both; yet, at optimal cut-off, its sensitivity as the MH marker was lower (Figure 4).

### 3.4. Nonenzymatic Serum Antioxidants and Inflammatory and Oxidative Stress Markers

Exclusively in patients with UC, SUA inversely but weakly correlated with IL-1 (ρ = −0.25, *p* = 0.035) and FT with TNF-α (ρ = −0.38, *p* = 0.044). In patients with active CD, FT correlated with hsCRP (ρ = −0.56, *p* < 0.001), ESR (ρ = −0.51, *p* = 0.003), PLT (ρ = −0.45, *p* = 0.008), and IL-6 (ρ = −0.45, *p* = 0.008). Solely FT, in patients with active CD, inversely correlated with AOPP (ρ = −0.45, *p* = 0.007), a protein marker of oxidative stress. TAS was negatively correlated with hsCRP concentrations regardless of IBD phenotype and the disease activity; yet, the relationship was the strongest in active CD (ρ = −0.56, *p* = 0.008) and UC (ρ = −0.60, *p* = 0.008). It was also inversely related to AOPP concentrations, although the relationship was statistically significant only in patients with UC (ρ = −0.38, *p* = 0.026).

In UC patients, TAS positively correlated with FT (ρ = 0.55, *p* = 0.007) and SUA (ρ = 0.49, *p* = 0.022) but there was no significant correlation between FT and SUA. In CD patients, TAS correlated with SUA (ρ = 0.68, *p* = 0.002) but not with FT and there was no significant correlation between SUA and FA.

### 3.5. Nonenzymatic Serum Antioxidants and Anemia

In active CD, but not UC, FT was significantly lower in patients with anemia (386 µM (349–423) vs. 467 µM (417–517), *p* = 0.009) and positively correlated with the concentrations of hemoglobin (ρ = 0.57, *p* < 0.001) or iron (ρ = 0.44, *p* = 0.016). SUA, solely in patients with active CD, was significantly lower in patients with anemia (2.5 mg/dL (2–3) vs. 3.5 mg/dL (2.9–4.1), *p* = 0.019) but only tended to correlate with hemoglobin (ρ = 0.30, *p* = 0.090) and iron (ρ = 0.32, *p* = 0.095). TAS was lower in patients with anemia, significantly so in UC patients (0.795 mM (0.721–1.316) vs. 1.365 mM (0.949–1.411), *p* = 0.038) but there was no significant correlation with the concentrations of hemoglobin or iron.

### 3.6. Nonenzymatic Serum Antioxidants and Treatment

FT was significantly lower in IBD patients treated with CS (400.9 μM (362–437) vs. 446.3 μM (429–477), *p* = 0.004). When accounting for the disease activity, significant differences were found between patients untreated with CS, both with inactive and active disease, who had the highest FT levels, and patients with active disease and treated with CS, who had the lowest FT levels. AZA treatment was not associated with significant changes in FT level (*p* = 0.183).

CS (*p* = 0.995) or AZA treatment (*p* = 0.880) had no effect on SUA. Similarly, there were no significant differences in TAS between IBD patients treated and untreated with CS (*p* = 0.182) or AZA (*p* = 1).

## 4. Discussion

Unclear and complex etiopathogenesis of inflammatory bowel disease results in the absence of a single diagnostic test and effective treatment. In the era of precision medicine, there is a need for further research toward the identification of biomarkers to improve IBD management strategies. Among many features of IBD, a lack of equilibrium between oxidants and antioxidants is repeatedly reported. Moreover, a potential of antioxidant therapies, which could block tissue damage and reverse natural history of IBD, is increasingly recognized [17,18,19]. In continuation of our study on oxidative stress in IBD, here, we demonstrated the diminished systemic nonenzymatic antioxidant capacity, in terms of thiol stress and reduced serum uric acid concentrations as well as total antioxidant status levels. We were able to do so, even though all our patients were treated with aminosalicylates, drugs displaying antioxidant activity [20] and used as a chemoprevention against cancer [21]. This finding supplements our earlier observations on reduced enzymatic antioxidants both in serum [9] and erythrocytes [10] and with systemic accumulation of lipid- [8] and protein-derived markers [7] of oxidative damage. It also corroborates reports on depletion of glutathione [22] and other low molecular-weight antioxidants, such as vitamins [22] and bilirubin [23,24].

An increased risk of colorectal cancer is a significant problem in IBD and IBD-associated oxidative imbalance in the colon might trigger neoplastic transformation [25,26]. In turn, systemic oxidative stress is likely to contribute to the development of the extraintestinal manifestations of the disease. These are common, affecting both CD and UC patients, and every second patient with IBD develops at least one extraintestinal manifestation which may be already present before IBD is diagnosed [27,28]. Accordingly, oxidative stress has been implicated in the pathogenesis of arthritis, ancylosing spondylitis [29], psoriasis [30], primary sclerosing cholangitis [11], liver cirrhosis, fatty liver [31], and anemia [32]. Anemia, which is the most common extraintestinal manifestation of IBD, results mainly from the combination of iron deficiency and anemia of chronic disease [33]. Oxidative stress is one of the proposed mechanisms responsible for the reduction of erythrocytes survival [32]. Here, we showed that thiol stress and SUA and TAS reduction are even more accentuated in IBD complicated by anemia, with thiol stress correlating with both hemoglobin and iron concentrations.

Our present study is focused on thiol stress, poorly addressed in IBD, and SUA, the status of which in IBD patients seems to be controversial, similarly to the role of uric acid in health and disease. Although recognized as a potent antioxidant, the blood elevation of uric acid above the norm is not desirable [34]. At high concentrations, uric acid forms crystals depositing in tissues and recent studies implicate even less severe hyperuricemia in the pathogenesis of metabolic disorders [34,35]. In contrast to viewing uric acid as a villain, an elevation in uric acid has been evolutionary favoured. Moreover, the fact that 90% of uric acid is recovered instead of being excreted with urine suggests its beneficial role. The most recognized benefit is associated with its antioxidant capacity. Uric acid is the most important serum antioxidant, claimed to scavenge over half of free radicals entering circulation [35]. Diminished SUA concentrations reported here, which reflect the disease activity by inversely correlating with CDAI in CD and by being significantly lower in active than nonactive UC, seem to imply that IBD is a condition in which the beneficial antioxidant activity of uric acid may prevail. The notion is further supported by an inverse correlation between SUA and Mayo endoscopic score, reflecting the severity of bowel inflammation. Considering the role of uric acid in IBD, one has also taken into account differences in the natural histories of IBD and conditions in which uric acid seems to play a negative role. The above-mentioned metabolic disorders are associated with overeating, improper diet and fructose overload, recognized as main causes of hyperuricemia [34,36]. These, however, are unlikely scenarios in IBD patients, who suffer rather from nutritional deficits [37]. Still, our observations do not seem to agree with recently published findings of Zhu et al. [38], who reported an increased retention of uric acid as indicated by elevated uric acid-to-creatinine ratio. Whether enhanced uric acid retention might be a compensatory mechanism meant to counteract oxidative imbalance has not been addressed. Moreover, their observation was restricted to patients with colonic CD and not with UC or noncolonic CD. Therefore, the discrepancy might be population-based, resulting from differences in proportion of colonic CD, azathioprine treatment and the way this drug is metabolized (effect of is discussed further), microbiome composition (affected by ethnicity and diet), and/or differences in methodology. However, it is worth mentioning that Zhu et al. [38] linked SUA elevation and retention with intestinal production of uric acid by *Saccharomyces cerevisiae*, supporting their thesis by demonstrating its positive correlation with anti-*Saccharomyces cerevisiae* antibody titer. Yet, the latter finding, implying a pathogenic role for these microorganisms in IBD, has not been confirmed by others [39]. Results on TAS are in favour of decreased SUA in IBD as uric acid is a key antioxidant component measured with various assays evaluating “total” antioxidant status/capacity (and referred to as, respectively, TAS or TAC). Therefore, in a way, diminished levels of TAS/TAC in IBD, found here as well as reported by others [40], corroborate the notion on decreased SUA in IBD. This is further supported by SUA and TAS correlation with the severity of bowel inflammation and the presence of extraintestinal manifestations of the disease.

Another important finding of our study is showing systemic thiol stress in IBD patients, manifested by decreased FT levels in both CD and UC patients, which parallel the severity of the disease expressed in terms of clinical activity indices and endoscopic findings as well as inflammatory indices and oxidative stress markers. It is worth to mention, that the significance of free thiols relies not only on their antioxidant activity but also on a key role played in redox signaling. Cysteinyl thiols are preferentially targeted by hydrogen peroxide, a secondary messenger, and, in signal transduction, their reversible oxidation to sufonic acid serves the same purpose as phosphorylation of serine, tyrosine, or threonine residues [41]. The relevance of having sufficient pool of free thiols for a cell can be demonstrated by the fact that drugs targeting FT are efficient bactericides [42]. Concerning systemic pool, FT are recently gaining attention as potential biomarkers in various pathologies [43,44,45,46]. The systemic FT pool consists of cysteinyl thiols of serum proteins available for oxidation, mostly albumin, and low molecular weight thiols, encompassing mostly glutathione and cysteine and its derivatives. Albumin might have a single free cysteine residue but by the virtue of its abundance in serum, it is crucial for maintaining the redox balance [47]. Due to nutritional deficiencies, especially in CD, albumin concentrations are frequently decreased in IBD patients. Moreover, hypoalbuminemia is a valuable predictor of the need for colectomy [48]. In our cohort, albumin was significantly lower in UC patients and significantly more so in CD patients (data not shown). This, by itself, might substantially diminish the FT pool. Here, we demonstrated that reduced albumin contributes to diminished FT pool; but, as the adjustment to albumin concentrations did not abolish FT association with IBD, the disease is accompanied by depletion of other FT components as well. Accordingly, aminoacid-based interventions restore local and systemic oxidative balance, leading to the alleviation of colonic damage and local and systemic inflammation in animal models of colitis (reviewed in [49]).

Simultaneously with the development of new therapeutic strategies, new treatment goals have been created, with mucosal healing being the most important. Currently, the golden standard to evaluate mucosal healing is invasive and expensive endoscopic examination [4,50]. Noninvasive, serum- or stool-based mucosal healing markers are intensively researched. In this respect, our findings on FT, SUA, and TAS decline correlating with severity of colonic inflammation, as expressed by increasing Mayo endoscopic score, is of clinical relevance. Therefore, we also examined FT, SUA, and TAS as potential markers of mucosal healing, showing superior accuracy of TAS. One of the limitations of our study was evaluation of the colonic mucosal healing only in ulcerative colitis patients. It would be interesting to assess FT, SUA, and TAS as potential markers of mucosal healing in Crohn’s disease. However, there was no representative group of CD patients with colonic location of the disease in our studied group and we were not able to perform this assessment.

An interesting finding of our study is significantly decreased thiol content in patients treated with corticosteroids. Corticosteroids are systemic, nonselective, anti-inflammatory drugs and the treatment with corticosteroids remains the mainstay of therapy for IBD flare [48,51,52]. Although highly effective and bringing immediate symptom relief, they are referred to as the “Dr. Jekyll and Mr. Hyde” of IBD therapy [52] due to severe side-effects and the fact that 20–25% patients become steroid-dependent [48]. Our results imply that exacerbation of systemic thiol stress may be added to the list of adverse effects of corticosteroids therapy. An anti-inflammatory effect of corticosteroids is associated with their inhibitory effect on immune cells, including impairing their cytotoxic function [52]. Therefore, one might expect corticosteroids to decrease reactive oxygen species (ROS) production by phagocytic cells and thus to alleviate the oxidative stress. However, unlike with monocytes, blood count of ROS-generating neutrophils transiently increases following corticosteroids intake [52]. Moreover, studies on corticosteroids-induced neurotoxicity have implicated oxidative stress as a leading mechanism. It has been shown that corticosteroids stimulate ROS generation by inducing the expression of NADPH-oxidase (NOX) [53]. It is worth mentioning that NOX expression is also upregulated by TNFα, GM-CSF, and PDGF [41], cytokines over-secreted in IBD and reflecting the severity of oxidative stress and mucosal inflammation [54,55]. To further tip the oxidative balance, corticosteroids downregulate the expression of enzymatic antioxidants [53]. Therefore, corticosteroids action, via increasing neutrophil count and NOX expression, is likely to translate into systemic elevation of hydrogen peroxide, the relatively stable ROS capable of membrane diffusion. Hydrogen peroxide preferentially reacts with cysteinyl thiols, rendering cysteine, glutathione, and protein thiol groups, of all blood antioxidants, the most susceptible for oxidation [41]. This phenomenon explains why only FT decrease was significantly associated with corticosteroids treatment in our study. Thiol stress is further aggravated by the fact that neutrophils, unlike monocytes/macrophages and dendritic cells, do not seem to co-secrete protective antioxidants (mainly cysteine) during their oxidative burst [41].

We also investigated the potential association between nonenzymatic antioxidants and azathioprine treatment. Azathioprine is an immunosuppressant, recommended for steroid-dependent IBD patients and to maintain remission [48]. Azathioprine may have the opposing effects on systemic antioxidants. On one hand, it has been shown that its cytotoxicity towards hepatocytes [56] or lymphocytes T [57] is mediated by drug-induced oxidative stress and accompanied by an elevation in systemic hydrogen peroxide [56]. Azathioprine-associated increase in systemic hydrogen peroxide is likely, as discussed earlier, to deplete systemic thiol [41]. On the other hand, however, azathioprine, which can undergo biotransformation via three different enzymatic systems, and if metabolized by xanthine oxidase, yields thiouric acid [58]. Thiouric acid is a compound sharing antioxidant capacity with uric acid but additionally equipped with free sulfhydryl group. Indeed, sulfur derivatives of uric acid have been shown to exert neuroprotective effects and lessen ischemic brain damage [58]. Therefore, these opposing effects of a drug might be the reason that the net effect observed in our patients was a lack of difference in both SUA and FT levels in patients treated and nontreated with azathioprine.

Even if our finding did not elucidate whether the problem of oxidative stress in IBD pathogenesis is “an epiphenomenon or the cause” [59], it should be noticed that there is a growing number of the experimental studies showing benefits from administration of antioxidants in colitis which fully justifies our interest in the oxidative stress in IBD. Oxidative/antioxidative balance restoration is listed next to stem cell transplantation or targeting the gut-associated lymphoid tissue as an “emerging nonconventional strategy in IBD therapy” [60].

## 5. Conclusions

IBD patients, regardless of the disease phenotype, have systemic thiol stress, depleted total antioxidant capacity, and reduced concentrations of uric acid, reflecting, to various degrees, clinical and local disease activity as well as presence of anemia, the most common extraintestinal manifestation of IBD. Evaluation of systemic total antioxidant status may be useful in noninvasive assessment of mucosal healing. Our findings on thiol stress provide an additional aspect of the adverse effects of corticosteroids therapy.

## Figures and Tables

**Table 1:** Characteristics of the control and study group according to sex, age, disease activity, medical therapies, and selected inflammatory and nutritional indices.

Parameter	Controls	CD Patients	UC Patients	*p*-Value
Number of cases	57	47	71	
Sex (F/M)	27/30	23/24	31/40	0.837 ^Χ2^
Age [years]	42 (35–50.4)	40 (30.6–42.7)	46 (36.1–50.2)	0.260 ^K^
Active disease, n (%)	-	37 (78.7)	30 (42.3%)	<0.001 ^F^
CS, n (%)	-	20 (42.6)	23 (32.4)	0.329 ^F^
AZA, n (%)	-	19 (40.4)	15 (21.1)	0.037 ^F^
BMI [kg/m^2^]	25 (21.7–28.2)	23.1 (21.4–24.7)	23.5 (22.4–24.6)	0.445 ^A^
ALB [g/dL]	4.77 (4.7–4.9) ^a,b^	4.23 (4.1–4.4) ^b,c^	(4.48 (4.4–4.6) ^a,c^	<0.001 ^A^
hsCRP [mg/L]	1.84 (0.84–4.95) ^a,b^	52.9 (17.4–99.3) ^c^	25 (13.8–43.5) ^c^	<0.0001 ^K^
HGB [g/dL]	13.2 (12.3–15)	12.1 (11.4–12.6)	12.8 (12.2–13.5)	0.084 ^K^
Iron [μM]	18.6 (15.7–21.4) ^a,b^	10.8 (8–12.5) ^b,c^	15.5 (13.4–17.6) ^a,c^	<0.0001 ^K^
ESR [mm/hr]	9 (7.2–15) ^a^	20 (16.8–26.5) ^b^	14.5 (11.7–22)	0.018 ^K^
PLT [×10^3^/mm^3^]	250 (216–320) ^a^	367 (330–411) ^b,c^	292 (276–316) ^a^	0.011 ^K^
WBC [×10^3^/mm^3^]	6 (5.47–6.61)	6.35 (5.6–8.16)	6.4 (5.8–7.53)	0.474 ^K^
IL-1 [pg/mL]	0.79 (0.31–1.71)	0.93 (0.59–1.37)	0.94 (0.7–1.41)	0.550 ^K^
IL-6 [pg/mL]	0.77 (0.68–1.14) ^a,b^	2.57 (1.83–3.84) ^c^	1.99 (1.23–2.82) ^c^	<0.0001 ^K^
TNF [pg/mL]	0.83 (0.4–1.44)	0.77 (0.37–1.04)	0.53 (0.35–0.94)	0.914 ^K^

If not otherwise stated, data presented as medians with 95% CI. and data on BMI and ALB as means with 95% CI. CD, Crohn’s disease; UC, ulcerative colitis; F, females; M, males; CS, corticosteroids; AZA, azathioprine; BMI, body mass index; ALB, albumins; hsCRP, high-sensitive C-reactive protein; HGB, hemoglobin; ESR, erythrocyte sedimentation rate; PLT, platelets; WBC, white blood cell; IL-1; interleukin-1; IL-6, interleukin-6; TNF, tumor necrosis factor; a, significantly different from CD; b, significantly different from UC; c, significantly different from controls; Χ2, Chi-square test; F, Fisher’s exact test; K, Kruskal-Wallis H test. A, one-way ANOVA.
